# Papillary lesions of the breast

**DOI:** 10.1007/s00428-021-03182-7

**Published:** 2021-11-03

**Authors:** Janina Kulka, Lilla Madaras, Giuseppe Floris, Sigurd F. Lax

**Affiliations:** 1 2nd Department of Pathology, Semmelweis University, Üllői út 93, 1091 Budapest, Hungary E.U.; 2 Department of Pathology, Uzsoki Hospital, Budapest, Hungary; 3 Department of Imaging and Pathology, Laboratory of Translational Cell & Tissue Research, KU Leuven, University of Leuven, University Hospitals Leuven, Leuven, Belgium; 4 Department of Pathology, Hospital Graz II, Graz, Austria; 5 School of Medicine, Johannes Kepler University, Linz, Austria

**Keywords:** Breast, Papillary lesions, Ductal carcinoma in situ, DCIS, Micropapillary, Biopsy

## Abstract

Papillary lesions of the breast represent a heterogeneous group of lesions including benign papillomas, papillomas with focal epithelial atypia, fully fledged ductal carcinoma in situ (DCIS) or lobular neoplasia, papillary DCIS, encapsulated papillary carcinomas without or with invasion, solid papillary carcinomas, and invasive papillary carcinomas. A micropapillary pattern characterized by lack of fibrous stalks within the papillae is observed in micropapillary DCIS and invasive micropapillary carcinoma. In addition, a variety of other rare breast lesions reveals a papillary architecture such as tall cell carcinoma with reversed polarity (TCCRP) and mucinous cystadenocarcinoma, adenomyoepithelioma, and secretory carcinoma. In addition, benign lesions such as usual ductal hyperplasia, apocrine metaplasia, gynecomastia, and juvenile papillomatosis may show a papillary or micropapillary architecture. Fragments of a benign papilloma in a breast biopsy are considered a lesion of uncertain malignant potential (B3 in the European classification) and excision is mostly recommended. Although the knowledge about molecular pathology of papillary breast lesions has increased, there is not sufficient evidence for diagnostically useful molecular features, yet. The aim of this review is to provide an update on papillary and micropapillary lesions with emphasis on problematic areas for daily diagnostic work including biopsies.

## Introduction

Diagnostic difficulties in the management of papillary breast lesions have been reflected by an increasing number of publications in recent years, including review articles covering the most relevant diagnostic aspects, molecular characteristics and management strategies [4, 10, 39, 64, 74, 88].

Papillary breast lesions are a clinically, histologically, and biologically heterogeneous group of breast diseases. Their main common histological feature is the presence of papillae mostly with arborising fibrovascular stroma. The formation of papillae is not a feature of normal breast tissue and the morphogenesis of papillary breast lesions is still not well understood [73]. It has been proposed that some papillary lesions result from a coordinated proliferation of stromal and epithelial cells, while in others the epithelial proliferation incorporates connective tissue of the involved duct’s wall [73]. When a papillary breast lesion is diagnosed the most important question is whether the lesion is benign, a precursor or malignant and in addition whether it is invasive or non-invasive. To rule out invasive growth, the presence of myoepithelial cells is basically important. In benign papillary lesions, myoepithelial cells are present together with luminal cells along the fibrovascular cores; however, myoepithelial cells may be absent or scant in benign apocrine papillomas and papillary apocrine hyperplasia [21]. The occurrence of cellular atypia, particularly in an associated ductal carcinoma in situ (DCIS), is accompanied by reduction and even lack of myoepithelial cells. In papillary DCIS, myoepithelial cells are present only at the periphery of the involved ducts. Encapsulated papillary carcinomas lack myoepithelium along the cyst wall, as well as do the nests of solid papillary carcinomas and of frankly invasive papillary carcinoma. Biologically, most carcinomas with papillary features are ER-positive and HER2 negative. Two recently acknowledged entities, tall cell carcinoma with reversed polarity and mucinous cystadenocarcinoma, are often triple negative but most cases show a relatively good prognosis [26, 42]. In contrast to usual papillae, micropapillae lack a fibrovascular core. The presence of micropapillae in breast lesions is less common but also of practical importance since a micropapillary pattern may be associated with various lesions such as usual ductal hyperplasia, DCIS, and invasive carcinoma.

The diagnostic problems of papillary breast lesions are reflected in the external quality assurance scheme of the UK National Health Service Breast Screening. Papillary lesions belonged to the most frequently misinterpreted breast lesions and were, particularly, over- or underdiagnosed based on HE sections alone [74]. Furthermore, the diagnosis of papillary lesions on core needle or vacuum assisted biopsy may be challenging. Even if fragments of a benign papillary lesion are found in a biopsy specimen, the presence of cellular atypia in another part of the lesion cannot be completely ruled out. This diagnostic uncertainty has led in Europe to the categorization of benign papillary lesions in biopsies as lesions of uncertain malignant potential or B3 on a 5 scale, regardless of the presence of cellular atypia [69]. In addition, the diagnosis of atypical epithelial proliferations on a biopsy may be challenging.

In this review, we would like to address clinical, radiological, and pathological features, and if available also molecular characteristics of the most important papillary and micropapillary breast lesions. Another focus is also diagnostic difficulties and dilemmas on core- or vacuum-assisted biopsies particularly for intraductal papillomas including B classification.

## Papillary neoplasms in the WHO classification of breast tumors

**Fig. 1:**
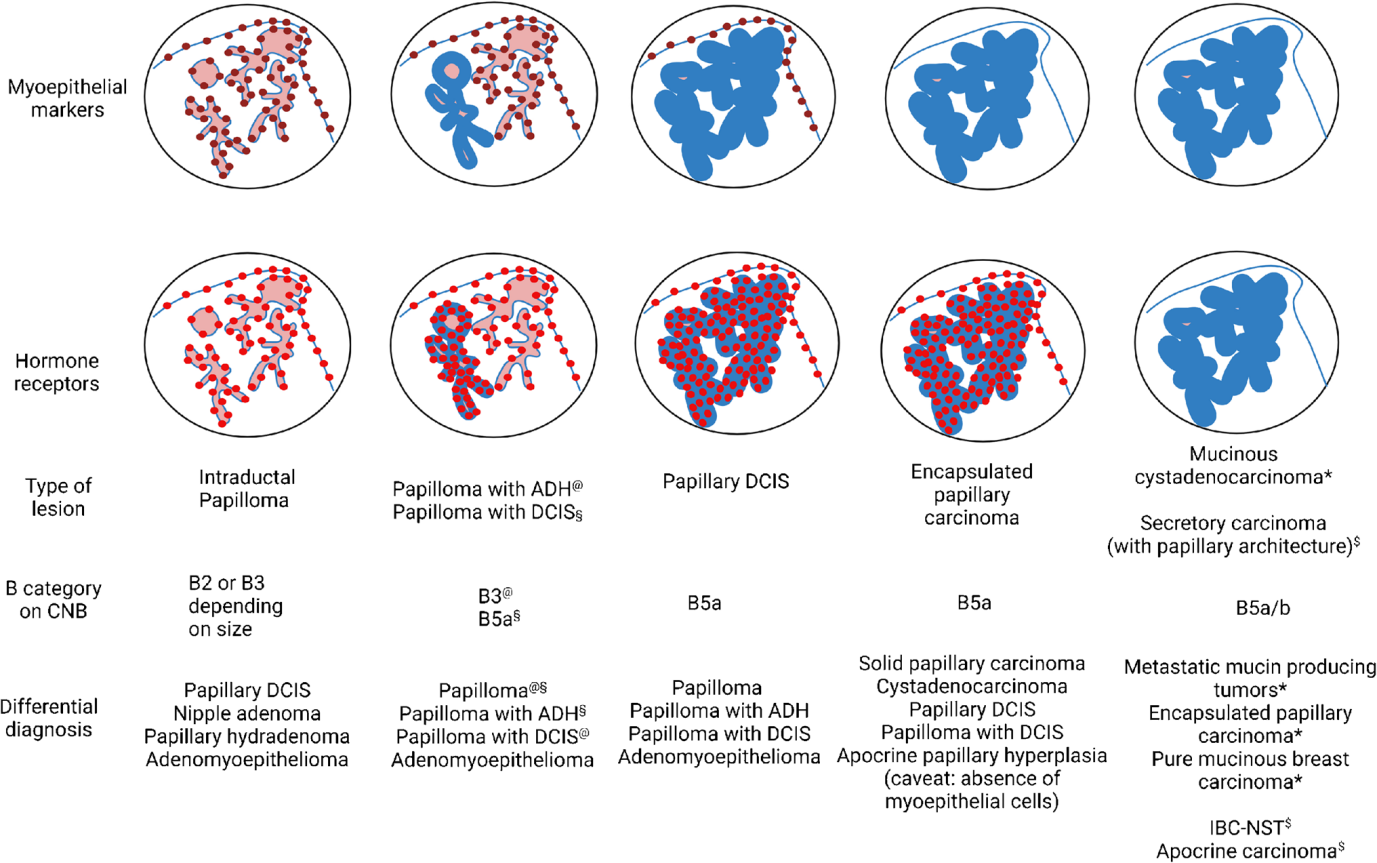
Staining patterns of myoepithelial markers and hormone receptors in various papillary lesions of the breast. The presence of myoepithelial markers is illustrated by bordeaux brown dots. Myoepithelial cells may be present or not in the peripheral wall and/or centrally in association with the branching fibrovascular cores illustrated in pink. In the same fashion, the bright red dots highlight the expression of hormone receptors in the lining epithelium. The lining epithelium is illustrated with a continuous blue line which depending on the degree of proliferation, and malignancy of the lesion becomes thicker with almost disappearance of the pink fibrovascular cores. Each combination of staining patterns is associated to specific lesions for which the B category on CNB, and the differential diagnosis is proposed. (This figure was created with BioRender.com)

Figure 1 provides an overview on papillary neoplasms listed by the 2019 WHO classification of breast tumors with emphasis on special features and differential diagnosis.

## Intraductal papilloma

Intraductal papillomas are benign intraluminal proliferations consisting of arborizing fibrovascular cores covered by a population of basal and luminal cells [85]. Intraductal papillomas are the most common papillary breast lesions and may be centrally or peripherally located. In a subset of cases, atypical epithelial proliferation may occur and is classified based on its extent as intraductal papilloma with atypical ductal hyperplasia (ADH) or with DCIS. Intraductal papilloma without atypical proliferation is also designated as NOS [85]. Most papillomas occur in perimenopausal women within an age range between 30 and 50 years. Central papillomas are more common than peripheral papillomas. They are not always identified on mammography. Larger lesions may appear as well-defined round or oval soft tissue opacities with or without microcalcifications. Ultrasound may reveal an intraluminal growth. Often, serous or sanguinolent nipple discharge is present. Galactography helps to identify the affected duct by showing filling defects caused by the intraductal growth. Peripheral papillomas are smaller, often multiple and usually asymptomatic. They may be associated with microcalcifications detected on mammography.

**Fig. 2:**
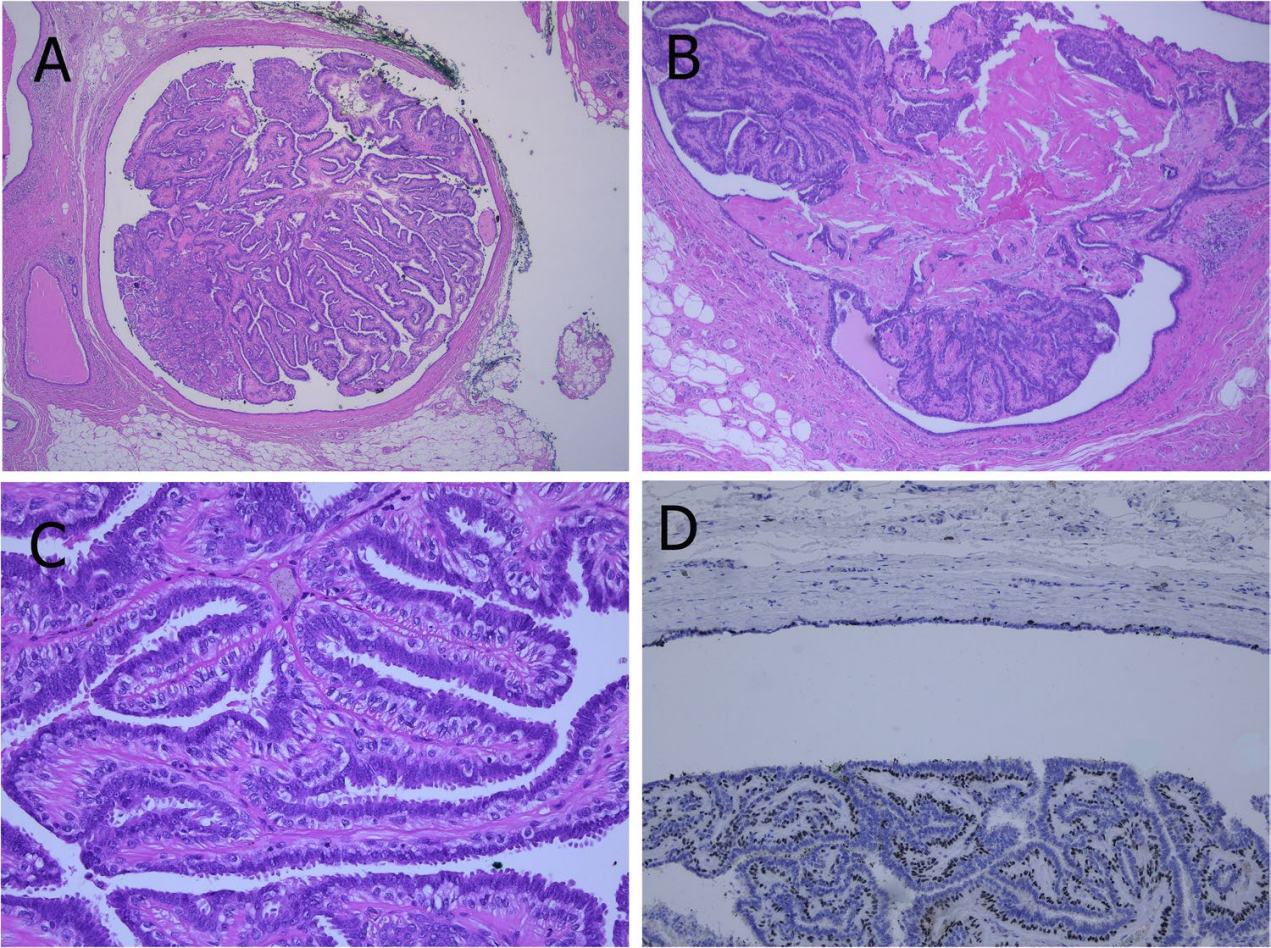
Papilloma in a large duct (central location) showing sclerotic areas (**A** and **B**). A prominent myoepithelial cell layer highlights the typical “two cell types” of benign papillary lesions (**C**). Myoepithelial cells can be demonstrated by p63 immunoreactivity both at the peripheral wall and along the fibrovascular cores (**D**)

**Fig. 3:**
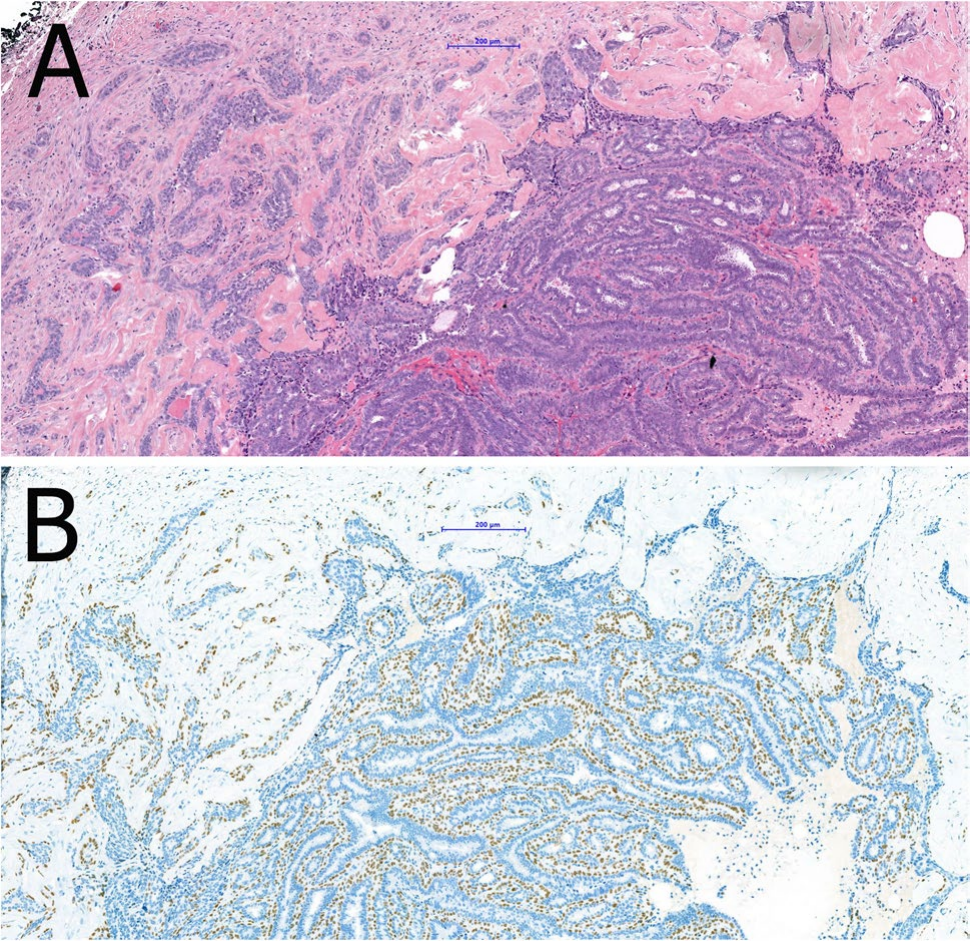
Sclerosis of the papilloma’s capsule with entrapped epithelial and myoepithelial cells may mimic a pseudo-infiltrative pattern (**A**). Myoepithelial markers such as p63 demonstrate the presence of myoepithelial cells and exclude invasion (**B**)

Histologically, complex arborizing fibrovascular cores lined by myoepithelial cells and covered by luminal cells are present within a dilated ductal space (Fig. 2). This evidence of two cell types is the hallmark for benign papillary lesions and absent in premalignant lesions [9]. The epithelial cells are either cuboidal or columnar, the nuclei may show intranuclear inclusions. In large papillomas, hemorrhage and infarcts may be present either due to prior needle biopsy or torsion of fibrovascular cores. Sclerosis and stromal fibrosis may imitate a pseudo-infiltrative pattern, and, particularly, in these cases myoepithelial markers may be very helpful (Fig. 3). Squamous, apocrine, mucinous, and chondroid metaplasia may occur and occasionally, collagenous spherulosis may also be present [85]. In peripheral papilloma, epithelial proliferation such as usual type ductal hyperplasia (UDH), ADH, atypical lobular hyperplasia (ALH), and DCIS is more common. High molecular weight cytokeratins (CK5, CK14) and heterogeneous positivity for estrogen receptor (ER) can be helpful to exclude atypical epithelial proliferation. Intraductal papillomas arising in the axillary tail need to be differentiated from rare sweat gland papillary hidradenoma. Care must be taken not to over-diagnose displaced epithelial elements of a papilloma into the surrounding breast parenchyma following fine needle aspiration- or core biopsy. The presence of hemosiderin, inflammatory cells, histiocytes, granulation tissue, or cellular scar tissue may be good indicators for an artifact. In the case of adenomatous growth pattern, adenomyoepithelioma may be considered. Differentiation from nipple adenoma may be challenging in cases of centrally located papilloma with sclerosing features. However, nipple adenoma arises mostly from the dermo-epidermal junction and less frequently from large ducts [85].

## Intraductal papilloma with ADH, DCIS, or lobular neoplasia

These lesions harbor a low nuclear grade atypical epithelial proliferation covering a part of the papilloma. In intraductal papilloma with ADH, this proliferation is limited to < 3 mm of extent, whereas in intraductal papilloma with DCIS, it spans ≥ 3 mm. The term “atypical papilloma” has not been adopted by the recent WHO classification. There are no specific clinical or imaging features. Suspicious microcalcifications may be found on mammography.

**Fig. 4:**
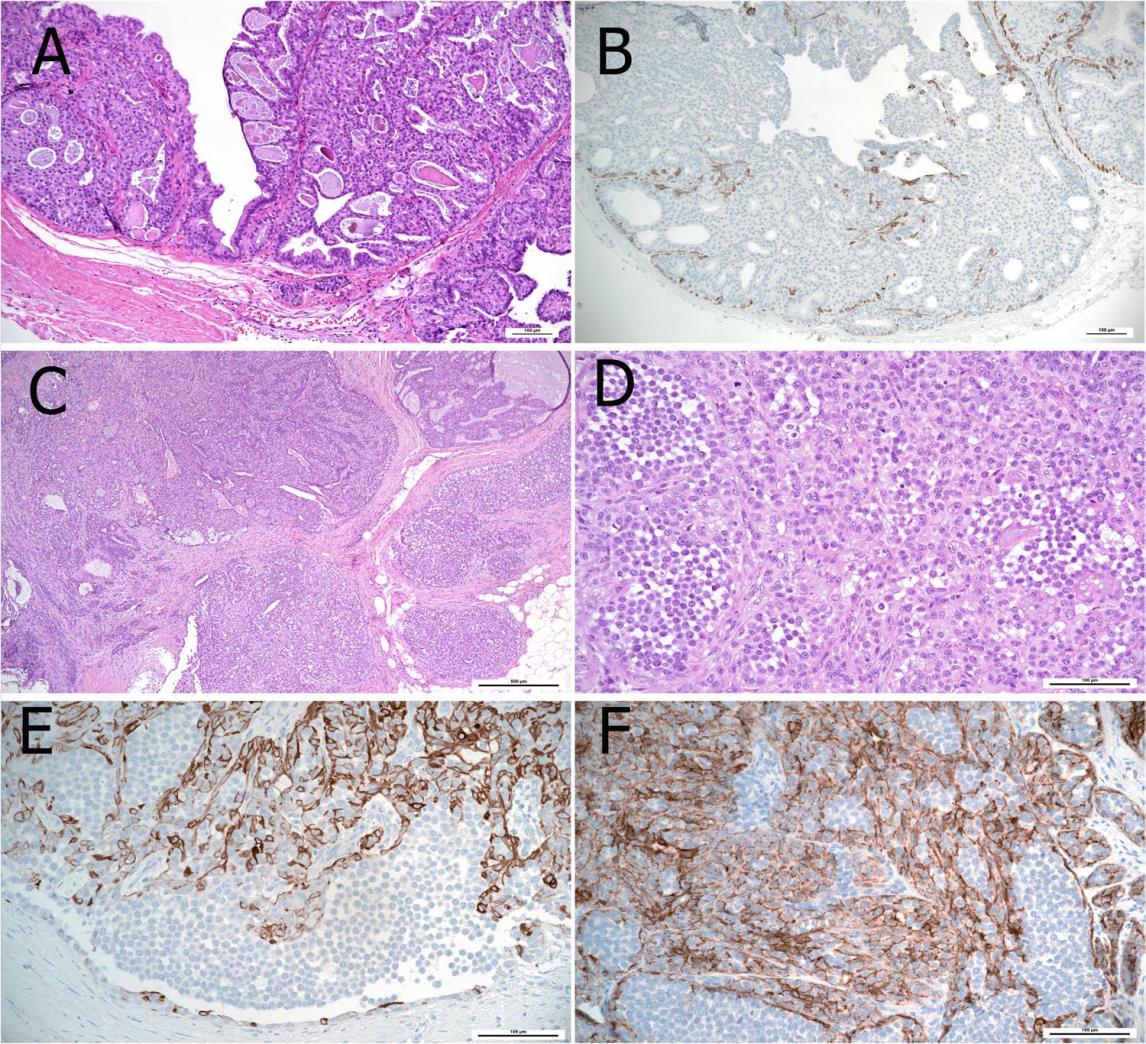
Intraductal papilloma with atypical epithelial proliferation of ductal type in an area of < 3 mm size qualifying for the diagnosis of ADH (**A**). The atypical epithelial proliferation lacks CK5 immunoreactivity (**B**). Intraductal papilloma with lobular neoplasia characterized by a solid proliferation of monomorphic cells with reduced cell cohesion (**C, D**). The lobular neoplasia is characterized by lack of immunoreactivity for CK5 (**E**) and e-cadherin (**F**)

The atypical ductal proliferation consists of cells with uniform, hyperchromatic nuclei often in cribriform arrangement (Fig. 4). Basally differentiated cells are not present. Immunohistochemistry underlines the neoplastic proliferation of luminal differentiated cells which are negative for high molecular weight keratins (CK5, CK14) and strongly and uniformly positive for ER. DCIS may be limited to the papilloma or may also involve the adjacent breast tissue [11]. If intermediate or high-grade atypia is present in a papilloma, the lesion should be classified as papilloma with DCIS regardless of the size of the atypical epithelial proliferation [11]. The risk of synchronously associated DCIS (or rarely that of invasive carcinoma) after diagnosis of papilloma in core needle biopsy is basically determined by the detection (or absence) of atypical epithelial proliferation [20]. The risk of “upgrade” of a papilloma without atypia after CNB in diagnostic excision is 2–3% [11]. The corresponding upgrade rate of a papilloma with atypical epithelial proliferation after CNB in diagnostic excision is 5.11%, and the upgrade rate after CNB with isolated atypical epithelial proliferation is 4.17% [46]. The risk of recurrence is more closely related to the presence of DCIS in the surrounding breast tissue than to the papilloma itself.

Less frequently, foci of lobular neoplasia may be present within an intraductal papilloma and this should be included in the pathology report (Fig. 4). E-cadherin and/or immunohistochemistry for catenin (p120 or β-catenin) may be helpful to highlight the area of lobular type atypia [11]. Intraductal papilloma with lobular neoplasia diagnosed on CNB or VAB does not require excision if radiological and pathological findings are concordant [11].

Earlier molecular studies suggested that alterations of *c-Met*, *RET/PTC*, *α3β1 integrin*, *Sonic hedgehog* (*Shh*), and *Bone Morphogenetic Protein* (*BMP*) play a role in the development of papillary breast lesions [71]. Recently, progression to DCIS and invasive carcinoma was hypothesized for a subset of intraductal papilloma [40]. Intraductal papillomas were clonally related to synchronous DCIS and invasive carcinoma in more than 50%, even without papillary histology of the latter. In pure intraductal papillomas, the most common finding was loss of chromosome X, followed by loss of 16q and 7q. The most common mutation was *PIK3CA* activating missense mutation. Increasing copy number alterations, especially 1q gain, 16q loss, and 11q loss, seem to result in progression. It was suggested that an intraductal papilloma without *PIK3CA* mutation could progress directly to papilloma with ADH/DCIS.

### Clinical management of intraductal papillomas detected on CNB and VAB

According to the European classification system, diagnostic CNB and VAB containing fragments of intraductal papillomas are coded as B3 and, due to the histomorphological heterogeneity of papillomas, excision is recommended [7, 45, 77]. However, the upgrade rate to DCIS and invasive carcinoma after surgery is low and varies between 0 and 16% (Table 1) [13, 15, 43, 49, 52, 58, 60, 62, 66]. Recent studies investigated upgrade rates and necessity of excision versus only imaging follow-up after biopsy. High risk lesions were found in 9.5% of 327 intraductal papillary lesions undergoing excision, DCIS in 3.4%, and invasive carcinomas in 2.4% [43]. Upgrade to DCIS or invasive carcinoma was more common among women over the age of 50 years, with lesions > 1 cm, lesions presenting as palpable mass, or if the lesion was > 5 cm distant from the nipple [43]. Among 61 patients under follow-up by imaging, no cancers were detected. In another recent study, features predicting upgrade were older age (median 64 versus 55), higher BIRADS category (≥ 4), lesion size (≥ 0.5 cm), and mass lesions with calcifications [52]. It was suggested that in particular, younger women with non-mass abnormalities and low BIRADS categories may benefit from clinical and imaging follow-up alone [52]. In a recent series analyzing symptomatic cases only, a general 2.4% upgrade rate was found with an upgrade to ADH and LN of 12.1%, but no predictive features for upgrade were identified [60]. It was suggested that if the whole lesion is removed by VAB and lacks atypia, there is no need for further surgery. No upgrade to malignancy was found if the benign papillary lesion was diagnosed on 11 G VAB and followed by excision [15]. Only the presence of atypia in a papilloma and older age were associated with upgrade to malignancy. Nevertheless, long-term follow-up is recommended [8]. In summary, these studies challenge the necessity of general excision of papillomas due to low upgrade rates. In 2018, the Second International Consensus Conference on lesions of uncertain malignant potential in the breast (B3 lesions) concluded that surveillance is appropriate for intraductal papillomas fully removed by VAB. Larger lesions which cannot be completely removed by VAB need open surgery and postoperative surveillance. In contrast, small papillomas (< 2 mm) may be coded as B2, if no atypia is present and if in toto removal can be safely diagnosed [69]. Since multiple (more than 5) papillomas were shown to bear a threefold increased relative risk for subsequent development of breast cancer, long-term follow-up is recommended after surgical removal. The relative risk increases to sevenfold, if multiple papillomas are associated with ADH or LN [46]. We would like to suggest to use the term “papilloma with atypical ductal epithelial proliferation (ADEP)” for biopsies and to restrict “papilloma with ADH/DCIS to excision specimens.

**Table 1:** Upgrade rate of intraductal papillomas in selected studies

1st author (year of publication)	Number of cases (specific features)		Biopsy device	Upgrade to in situ or invasive (%)	Upgrade to a high-risk lesion (ADH, ALH, FEA, cLCIS) %	Conclusion	Suggestion
Chang (2011) [14]		49	11 G vacuum or core	0	6.1	Papillary lesions without atypia can be diagnosed accurately by US-guided vacuum-assisted biopsy	Surgical excision may not be required for IDP diagnosed by US-guided 11-gauge vacuum assisted biopsies
Pareja (2016) [62]		166 (lesions without atypia)	core	2.3	0 (papilloma with ADH were exclusion criterium)	Upgrade rate at the excision was low for IDP with radiological-pathological concordance	Conservative approach is appropriate for IDP without atypia on CNB and with concordant pathological-radiological assessment, regardless of size
Kuehner (2019) [41]		327 (mass lesions)	core	5.8	9.5	Overall outcomes for BPBLs diagnosed on IGCNB are favorable whether immediate surgical excision or imaging surveillance is the final treatment choice	Conservative approach is reasonable in the management of BPBLs diagnosed on IGCNB
Moynihan (2019) [57]		124 (symptomatic)	core	2.4	12.1	Low risk of upgrade to malignancy for patients with a diagnosis of IDP without atypia on CNB	Observation may be a safe alternative to surgical excision in selected cases
MacColl (2019) [49]		180 (multi-institutional; lesions without atypia)	core	12 (cLCIS also included)	0 (cLCIS included into upgrade)	Risk factors associated with invasive carcinoma are advanced patient age, high BI-RADS score. For radiologically identified lesions, higher risk for carcinoma is associated with size > 0.5 cm and calcifications	Younger women with biopsies targeting non-mass abnormalities and low BI-RADS scores may benefit from clinical and imaging follow-up alone
Nakhlis (2020) [59]		85 (multi-institutional; asymptomatic)	core	1.7	13	Very low upgrade rate to invasive cancer or DCIS on excision if IDP without atypia diagnosed on core biopsy of BI-RADS4 lesions	Routine excision is not indicated for IDP without atypia on CNB and with concordant imaging findings
Moseley (2021) [55]		102	9–18 G core ± vacuum	2.9	7.8	Personal history of breast cancer and lesion size are associated with upgrade to carcinoma	Follow-up by imaging at 6 months interval for 2 years in selected low-risk patients with no history of breast cancer, no clinical symptoms, and size < 1 cm
Lin (2021) [47]		165	core	3	N/A	The surgical upgrade rate for pure IDP on CNB in younger women is only 3%	The low upgrade rate should be part of the management discussion

Abbreviations: *ADH*, atypical ductal hyperplasia; *ALH*, atypical lobular hyperplasia; *FEA*, flat epithelial atypia; *cLCIS*, classical LCIS; *US*, ultrasound; *IDP*, intraductal papilloma; *CNB*, core needle biopsy; *BPBL*, benign papillary breast lesion; *IGCNB*, image guided core needle biopsy

## Papillary ductal carcinoma in situ

Papillary ductal carcinoma in situ (DCIS) is a rare subtype of DCIS with papillary architecture completely lined by neoplastic ductal epithelium. Like other subtypes of DCIS, papillary DCIS is a segmental disease and involves small or large ducts in central and peripheral locations. It is usually detected on mammography due to associated microcalcifications or the presence of nodular densities.

**Fig. 5:**
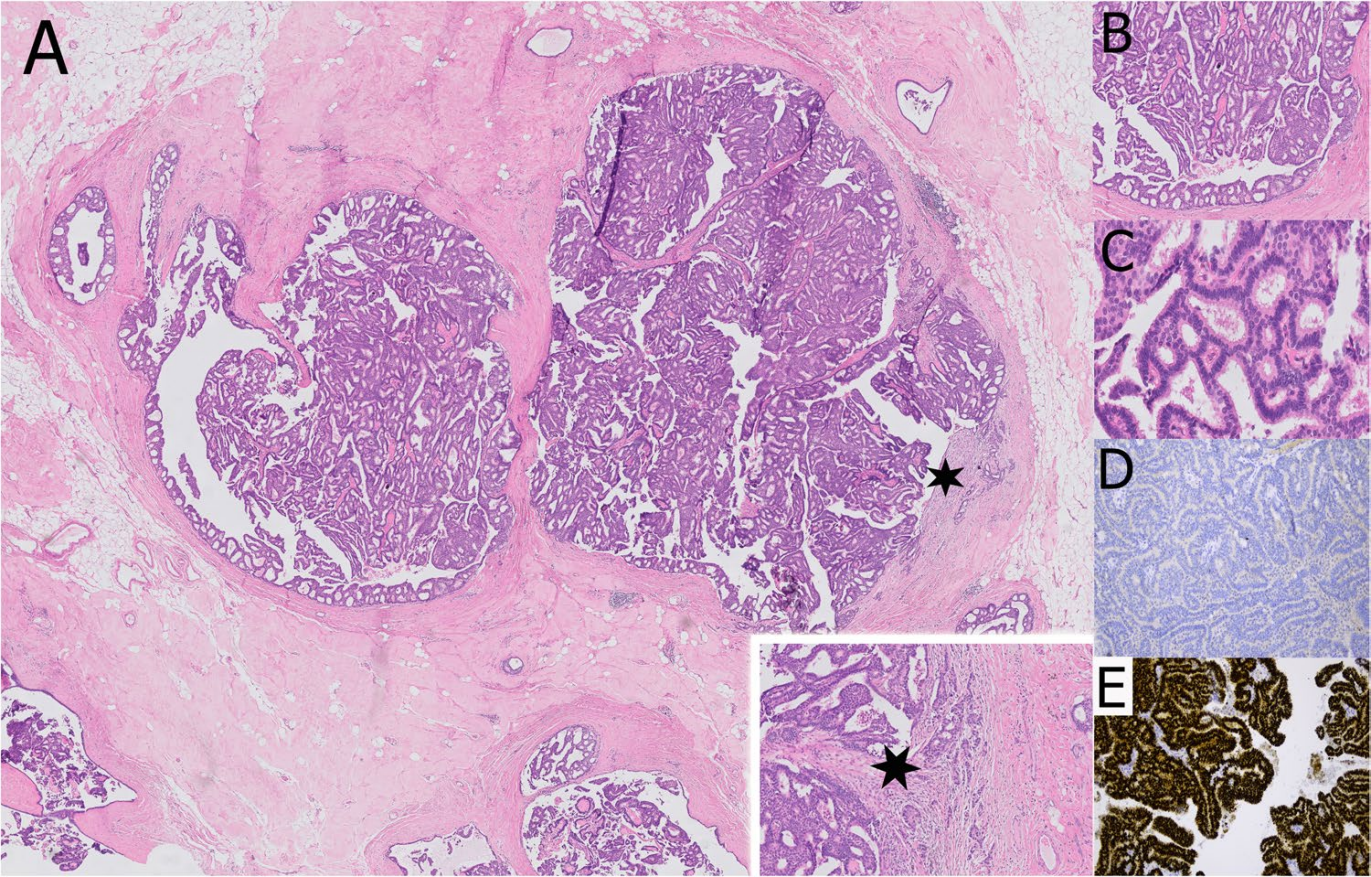
Papillary DCIS (**A**) with microinvasion (*). Cribriform architecture is present at the periphery of involved ducts (**B**). Characteristic features are low-grade nuclei (**C**), lack of myoepithelial cells as demonstrated by p63 (**D**) and diffuse, strong ER positivity (**E**)

The neoplastic epithelium is monomorphic and composed of one or several layers usually of columnar cells covering delicate branching fibrovascular cores (Fig. 5). Solid, cribriform, and micropapillary areas may be present. Myoepithelial cells are only present at the periphery of the ducts. The nuclear grade is usually low or intermediate. There is no evidence of pre-existing benign intraductal papilloma [20]. Papillary DCIS usually occurs together with other DCIS patterns, pure papillary DCIS is rare. A peculiar dimorphic variant has been described showing so-called “globoid cells” that can be mistaken for myoepithelial cells but are negative for all myoepithelial markers in immunohistochemistry [20]. Studies describing molecular alterations of pure papillary DCIS are scarce. They share some genetic alterations found in low-grade DCIS of other architecture. Studies of papillary breast lesions including invasive papillary carcinomas revealed LOH at loci 16q12.2, 16q21, and 16q23, but LOH at the *TP53* locus only in malignant papillary lesions [24, 93].

## Encapsulated papillary carcinoma

Encapsulated papillary carcinoma (EPC) is a tumor characterized by pushing borders and a papillary, cribriform or solid growth within a cyst. EPC usually presents as a slowly growing, indolent palpable mass with bloody discharge in postmenopausal women, rarely in men. Imaging commonly reveals a well-circumscribed, round, or oval retro-areolar lesion. The disease course is indolent with exceptional occurrence of axillary lymph node metastases [61, 75].

**Fig. 6:**
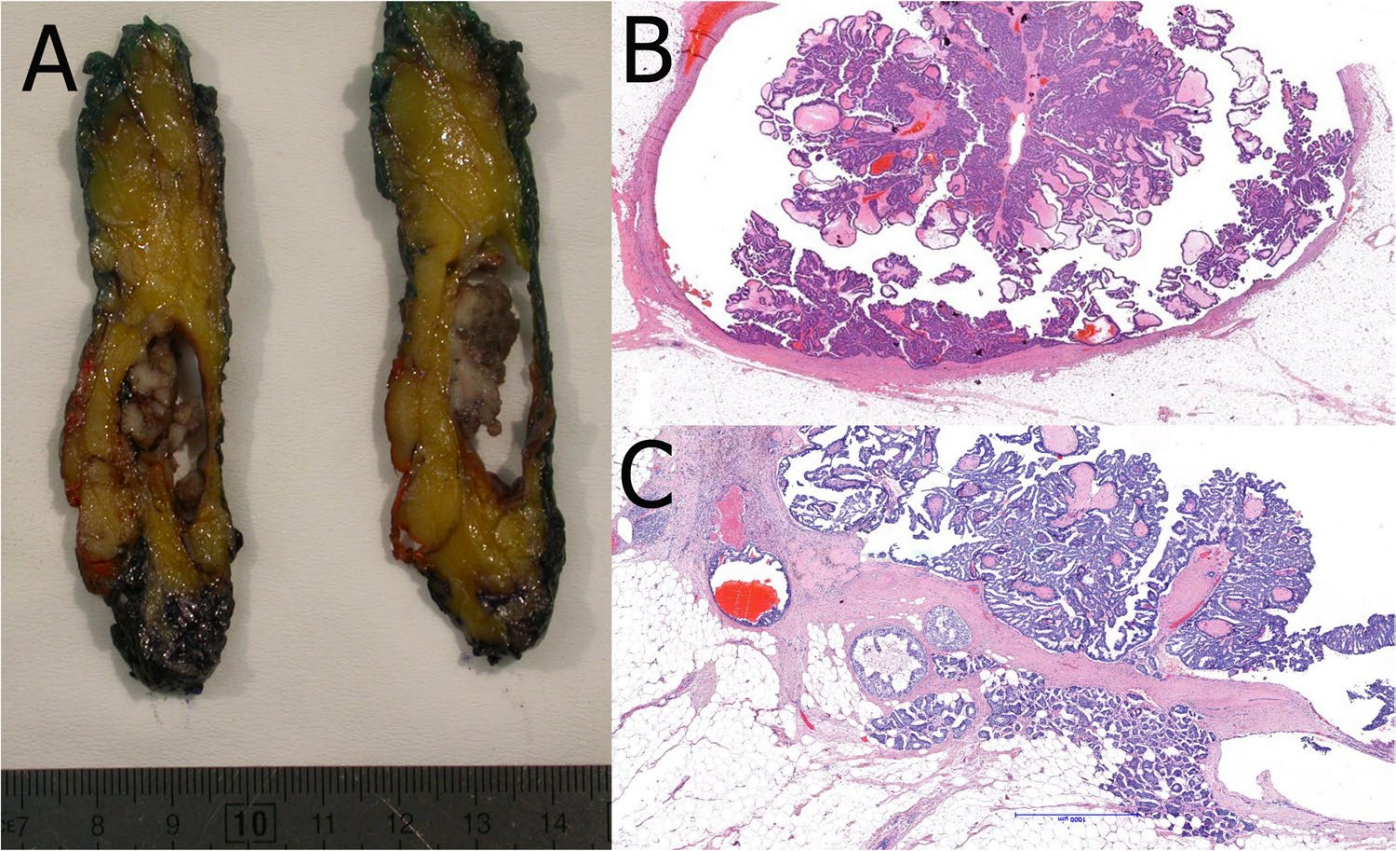
Encapsulated papillary carcinoma with typical gross appearance (**A**). An arborizing papillary structure lined by low-grade atypical epithelium is present within a cystically dilated space surrounded by a fibrotic capsule (**B**) and may be associated with frank invasion (**C**)

EPC consists of monomorphic cells with low to intermediate grade nuclei covering fine fibrovascular cores or occasionally forming cribriform or micropapillary structures (Fig. 6) [50]. EPC usually lacks myoepithelial cells in the papillae and at the periphery that suggests the possibility of an expansile growth pattern [19]. Infrequently, an incomplete myoepithelial cell layer may be seen [92]. EPC is usually surrounded by a thick fibrous capsule, sometimes with entrapped tumor cells. The tumor cells are usually ER and progesterone receptor (PR) positive and lack HER2 amplification. Low or intermediate nuclear grade DCIS, usually with micropapillary or cribriform architecture, may be seen in the surrounding breast tissue. EPC may be associated with invasive NST carcinoma, less frequently cribriform, mucinous, or tubular carcinoma, beyond the capsule. In the absence of a frank invasive carcinoma, EPC should be staged and managed as DCIS [41]. Those rare tumors with expansile growth pattern and papillary architecture, but high nuclear grade features and high mitotic activity should be staged and managed as invasive breast cancer [76].

Genomic characterization of EPC revealed frequent *PIK3CA* mutations similar to low grade, ER-positive invasive breast carcinomas [25]. By PAM 50, the majority of EPC is classified as luminal A tumors and only a small number as luminal B. Furthermore, EPC seems to differ from solid papillary carcinoma and invasive papillary carcinoma by downregulation of genes related to cell migration [68].

## Solid papillary carcinoma

Solid papillary carcinoma (SPC) is characterized by a solid growth pattern with delicate fibrovascular cores. Most SPC are unifocal and well circumscribed. They often are centrally located and cause nipple discharge. The prognosis is excellent with rare recurrence and only exceptional death of disease.

**Fig. 7:**
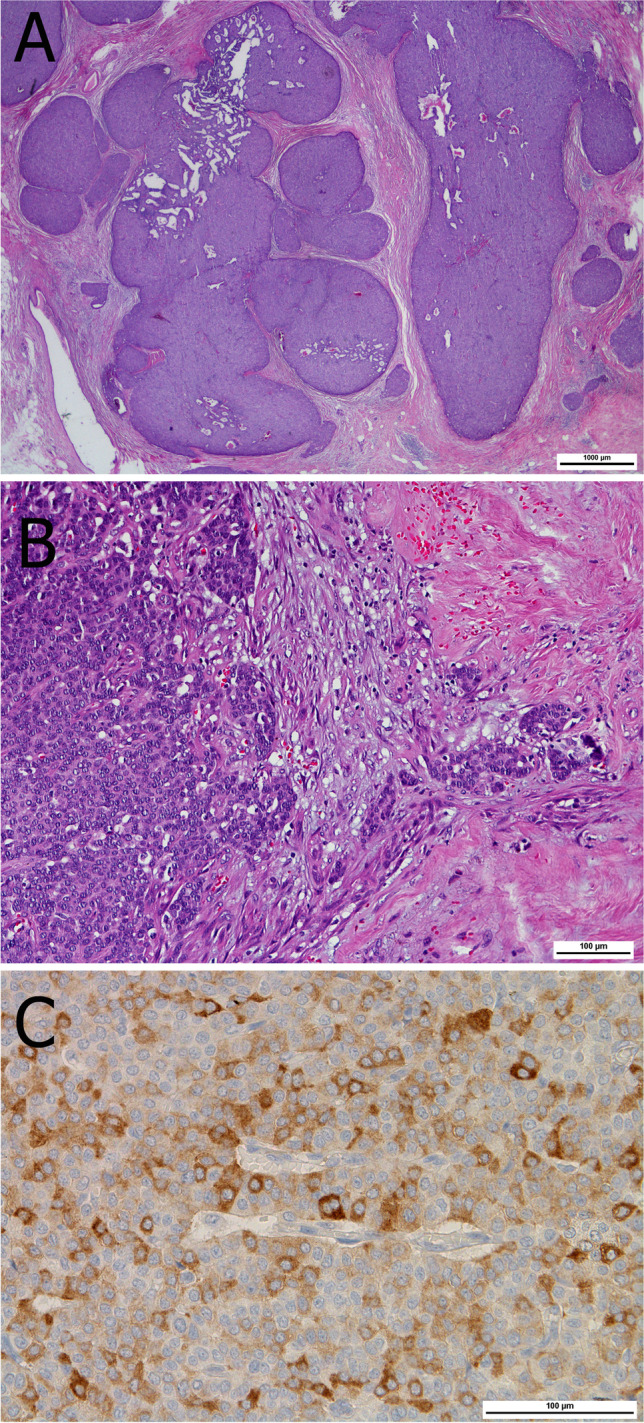
Solid papillary carcinoma characterized by a low-grade atypical epithelial proliferation with solid growth pattern filling dilated ducts (**A**). Invasion is characterized by small infiltrative ducts and nests (**B**). Neuroendocrine differentiation is frequently found as confirmed by expression of synaptophysin (**C**)

The histological features were described as in situ solid growth pattern filling large or dilated small ducts showing delicate fibrovascular septa that are sometimes sclerotic and nuclear palisading at the stromal epithelial interface (Fig. 7). Small- to moderate-sized cells with commonly round to ovoid or sometimes spindle-shaped, mildly atypical nuclei, and eosinophilic and granular cytoplasm are arranged in rosette or pseudo-rosette formations [53]. Neuroendocrine differentiation and mucin production are very common [65]. ER is diffusely and strongly positive. The complete absence of myoepithelial cells should not prevent from considering these tumors as in situ disease in presence of microscopic findings consistent with DCIS (e.g., rounded well-circumscribed structure in an organoid pattern). Rarely, tumors with features of SPC may show frank invasion and should be classified as invasive; this may be associated with a jigsaw pattern and a desmoplastic stromal response [51]. Invasion may also be associated with mucinous differentiation or present as carcinoma of no special type (NST) [51]. Invasive lobular carcinoma (ILC) mimicking SPC has been described in the differential diagnosis to SPC and also EPC but the small number of reported cases allows only limited conclusions [72]. Importantly, SPC-like ILC is a frankly invasive tumor [59].

Molecular studies included only a limited number of cases. No differences in copy number alterations were found between SPC, EPC, and invasive papillary carcinoma [68]. However, genes related to neuroendocrine differentiation (*RET*, *ASCL1*, and *DOK7*) were upregulated in SPC compared to EPC. Interestingly, all 4 cases analyzed by PAM50 were assigned to the luminal B subtype [68]. In another study using Oncotype DX, all SPC were associated with low and intermediate recurrence score (RS) [86]. One case of SPC-like ILC revealed an ILC-like molecular profile and a unique *CDH1/E-cadherin* mutation [17].

## Invasive papillary carcinoma

**Fig. 8:**
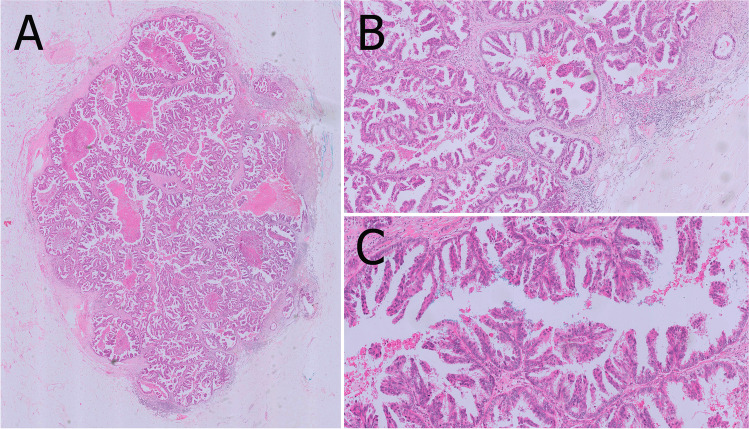
Invasive papillary carcinoma characterized by cystically dilated spaces of various size (**A**) and frank invasion with focal necrosis at the periphery (**B**). Delicate arborizing papillary structures are readily appreciated at medium power magnification (**C**)

Invasive papillary carcinoma is a very rare subtype of invasive breast carcinomas consisting of papillae with a fibrovascular core. There are no specific clinical or imaging findings. The papillae are located in dilated ducts and microcysts and lack myoepithelial cells at the periphery (Fig. 8). Tubules may be present. The nuclei are usually low grade with low to moderate number of mitoses. Invasive papillary carcinoma needs to be distinguished from invasive micropapillary carcinoma and other papillary tumors of the breast as well as from metastases of carcinomas with a papillary pattern (see below).

## Other breast tumors with papillary architecture

### Tall cell carcinoma with reversed polarity

Tall cell carcinoma with reversed polarity (TCCRP) has been formerly known as breast tumor resembling the tall cell variant of papillary thyroid carcinoma and as solid papillary carcinoma with reverse polarity. TCCRP is a rare subtype of invasive breast carcinoma consisting of tall columnar cells with reversed nuclear polarity arranged in a solid pattern. TCCRP usually presents as a well-circumscribed mass, measuring up to 5 cm in diameter.

**Fig. 9:**
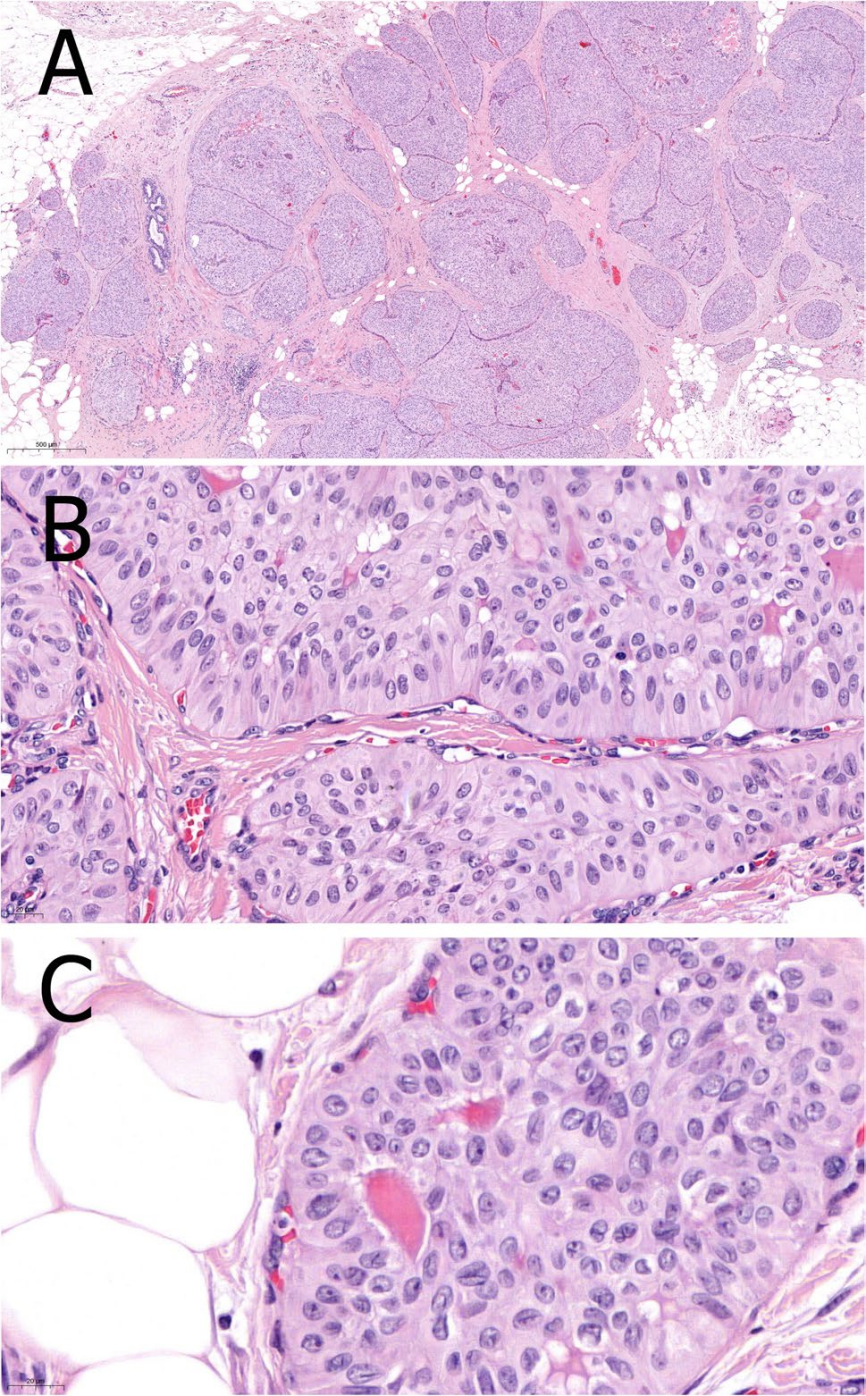
TCCRP showing multinodular architecture with presence of fibrovascular cores centrally located in the nodular structures (**A**). Narrow fibrous septae (**B**) and occasionally colloid-like eosinophilic material (**C**) are characteristic features

The close resemblance to papillary thyroid carcinomas results from its frequent demonstration of papillae and follicular structures, even with colloid-like material, psammoma bodies, tumor cell nuclei with grooves and inclusions (Fig. 9). The tumor cells are tall columnar with prominent eosinophilic cytoplasm rich in mitochondria. The nuclei are located at the apical areas of the cells hence the “reversed polarity” appearance. Myoepithelial cells are almost always missing. Foamy macrophages are often present within the fibrovascular cores. TCCRP is usually triple negative or weakly ER/PR-positive but with low Ki67 labeling index. Immunohistochemistry for calretinin is usually positive and negative for synaptophysin, chromogranin A, TTF1 and thyroglobulin. GATA3, GCDFP15, and mammaglobin are variably positive [5, 83].

A characteristic hotspot mutation R172 in the *IDH2* gene, which is otherwise rare in breast carcinomas, was detected in the vast majority of TCCRPs. Mutant *IDH2* can be detected by immunohistochemistry using a specific antibody [94]. Other tumors harbor *PRUNE2* mutations. Missense mutations affecting *PIK3CA* or *PIK3R1* may also be detected. *BRAF* mutations are not encountered.

### Mucinous cystadenocarcinoma

**Fig. 10:**
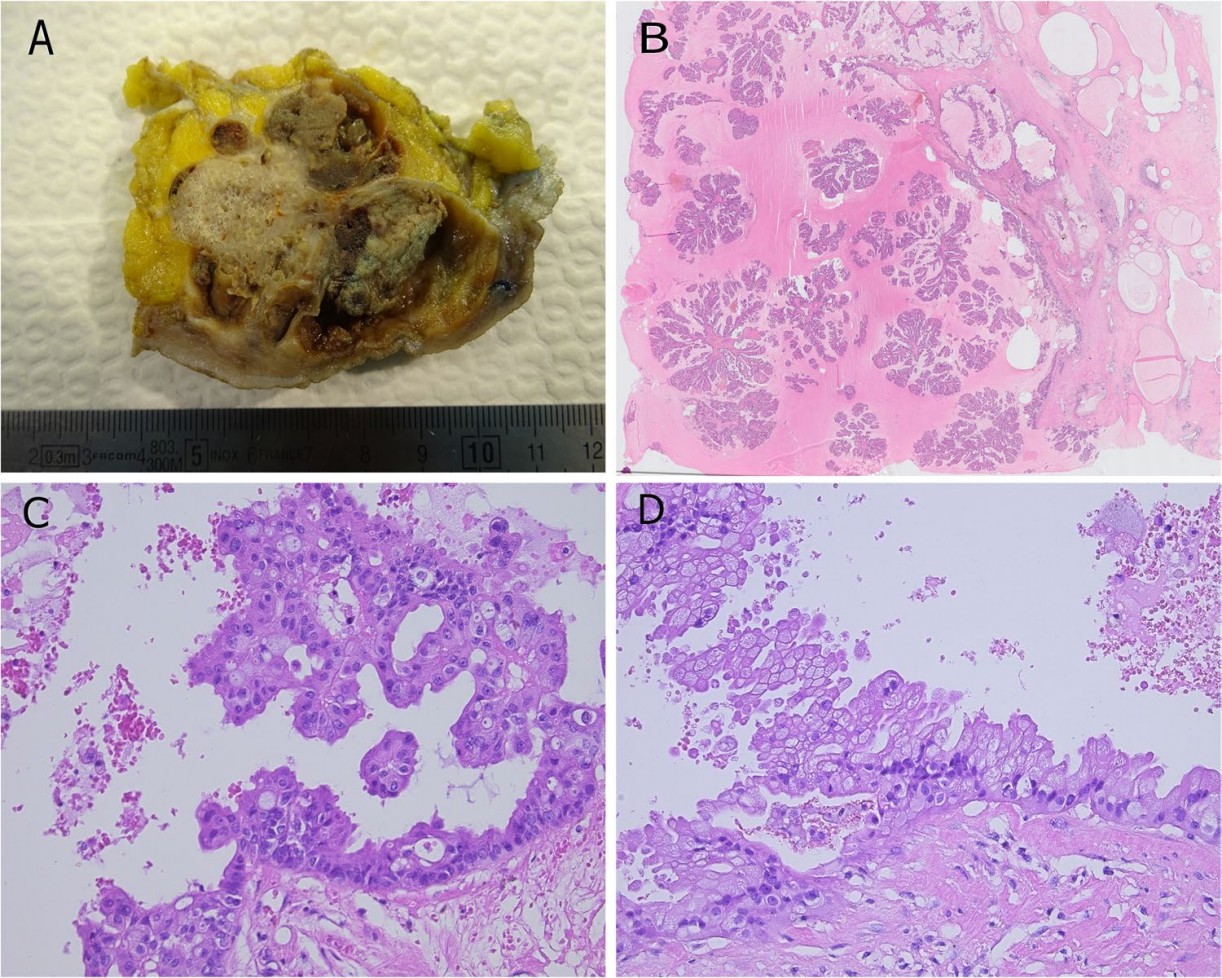
Mucinous cystadenocarcinoma consisting of cysts filled with abundant mucin (**A**). The delicate fibrous septa are covered by eosinophilic tumor cells with floating cellular morules on top (**C**). The tumor cells may show abundant mucin production (**D**)

Mucinous cystadenocarcinoma (MCA) is a very rare cyst-forming invasive breast carcinoma with papillae and abundant extracellular mucin (Fig. 10). So far, less than 35 cases have been published with predominance of Asian women [90]. The low number of cases could be explained by under-recognition. MCA usually occurs in postmenopausal women as a palpable mass with a relatively large diameter being ≥ 4 cm in 50% of the cases [38]. MCA is well circumscribed and often hypoechoic on ultrasound. The typical gross appearance is a gelatinous cyst. A prominent papillary architecture with swollen fibrovascular cores is often present and the papillae show hierarchical organization. An association with mucocele-like structures is frequent. Tumor cells are columnar with basally located nuclei, tufting, stratification, and abundant mucin production. Squamous morules or floating micropapillary groups may be observed at the tip of papillary projections and are considered a useful microscopic clue to distinguish MCA from other mucinous carcinomas of the breast [82]. Nuclear atypia is variable within the same lesion [42]. When DCIS is absent, metastatic origin should be ruled out because of overlapping features with pancreatic, appendiceal, and ovarian mucinous neoplasms. A panel of immunohistochemical markers including CK7, CK20, CDX2, and GATA3 is helpful. In contrast with pure mucinous carcinoma and EPC, which typically express ER and PR, MCA is triple negative [35, 90]. The majority of MCA has been treated by radical mastectomy; data regarding the need of systemic adjuvant therapy are missing. Lymph node involvement is rare in MCA and prognosis is considered favorable. However, the low level of evidence, as well as the lack of large case series with a long-term follow-up, recommends caution regarding therapeutic conclusions [38].

### Metastases to the breast with papillary architecture

Metastases from ovarian carcinomas, mucinous tumors of the GI tract, particularly the pancreas, renal cell carcinomas, lung adenocarcinoma, papillary thyroid carcinoma, and prostate ductal adenocarcinoma may show a papillary architecture and mimic a primary papillary neoplasm of the breast [44]. A case of metastatic gastrinoma mimicking SPC was reported [12]. Immunohistochemistry is helpful for differential diagnosis and may be crucial for certain cases.

### Micropapillary DCIS

Micropapillary DCIS (MP DCIS) is a pattern of DCIS characterized by the formation of micropapillae. In contrast to papillae, micropapillae lack a fibrovascular core. MP DCIS is frequently associated with “snake skin-like” microcalcification [81]. High rate of recurrence following breast conserving surgery has been reported [14].

**Fig. 11:**
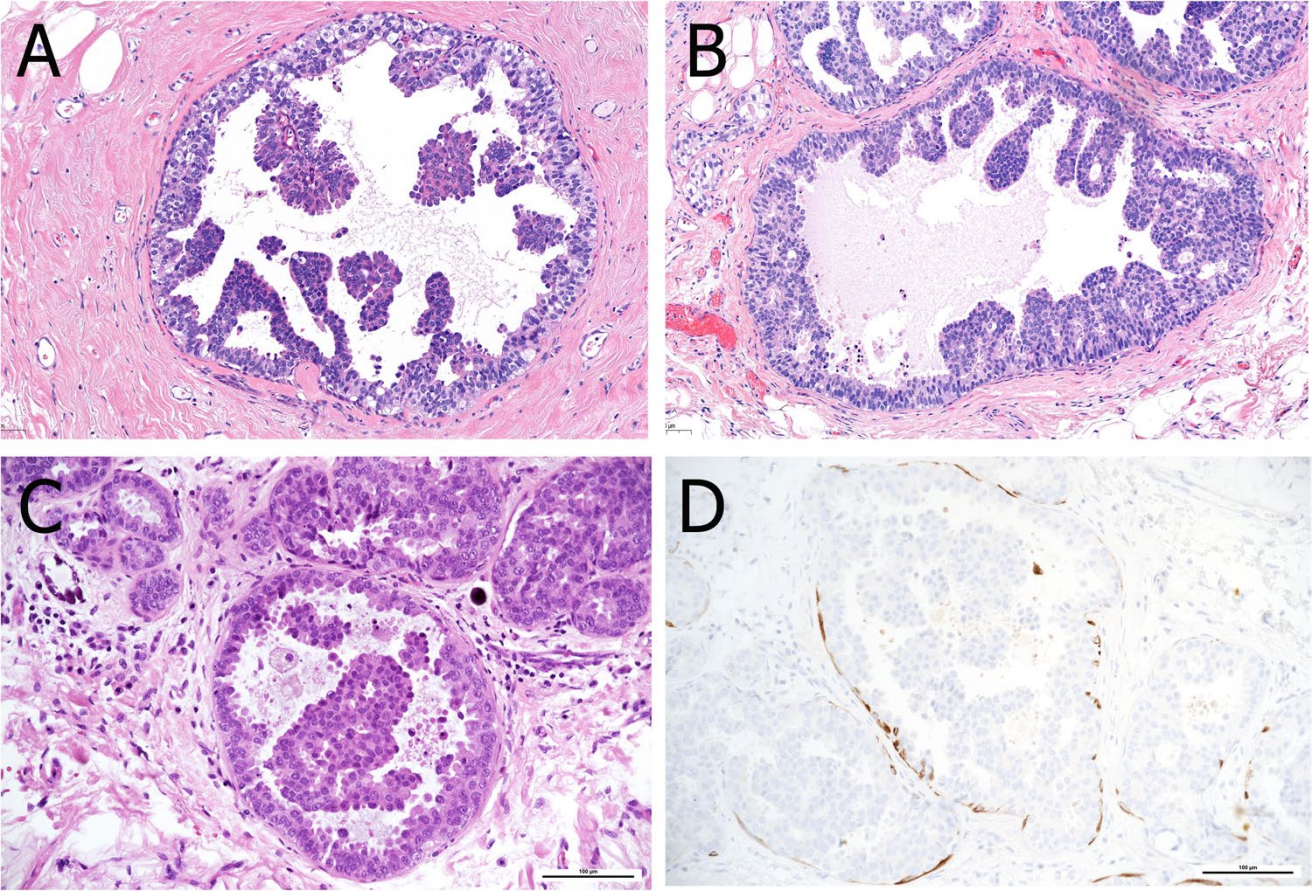
Micropapillary and papillary DCIS in a small duct (**A**) adjacent to invasive papillary carcinoma (not shown). Pure micropapillary DCIS is characterized by micropapillary projections lacking fibrovascular cores and typical triangular architecture observed in micropapillary hyperplasia (**B, C**). Immunohistochemistry for CK5 shows the lack of basally differentiated cells (**D**)

MP DCIS is composed of low-grade neoplastic cells involving usually small and mid-sized ducts (Fig. 11). MP DCIS may occur together with other patterns of DCIS (e.g., cribriform, solid). Immunohistochemistry with high molecular weight keratins (CK5 or 14) is useful to distinguish MP DCIS from UDH with micropapillary pattern.

### Invasive micropapillary carcinoma

**Fig. 12:**
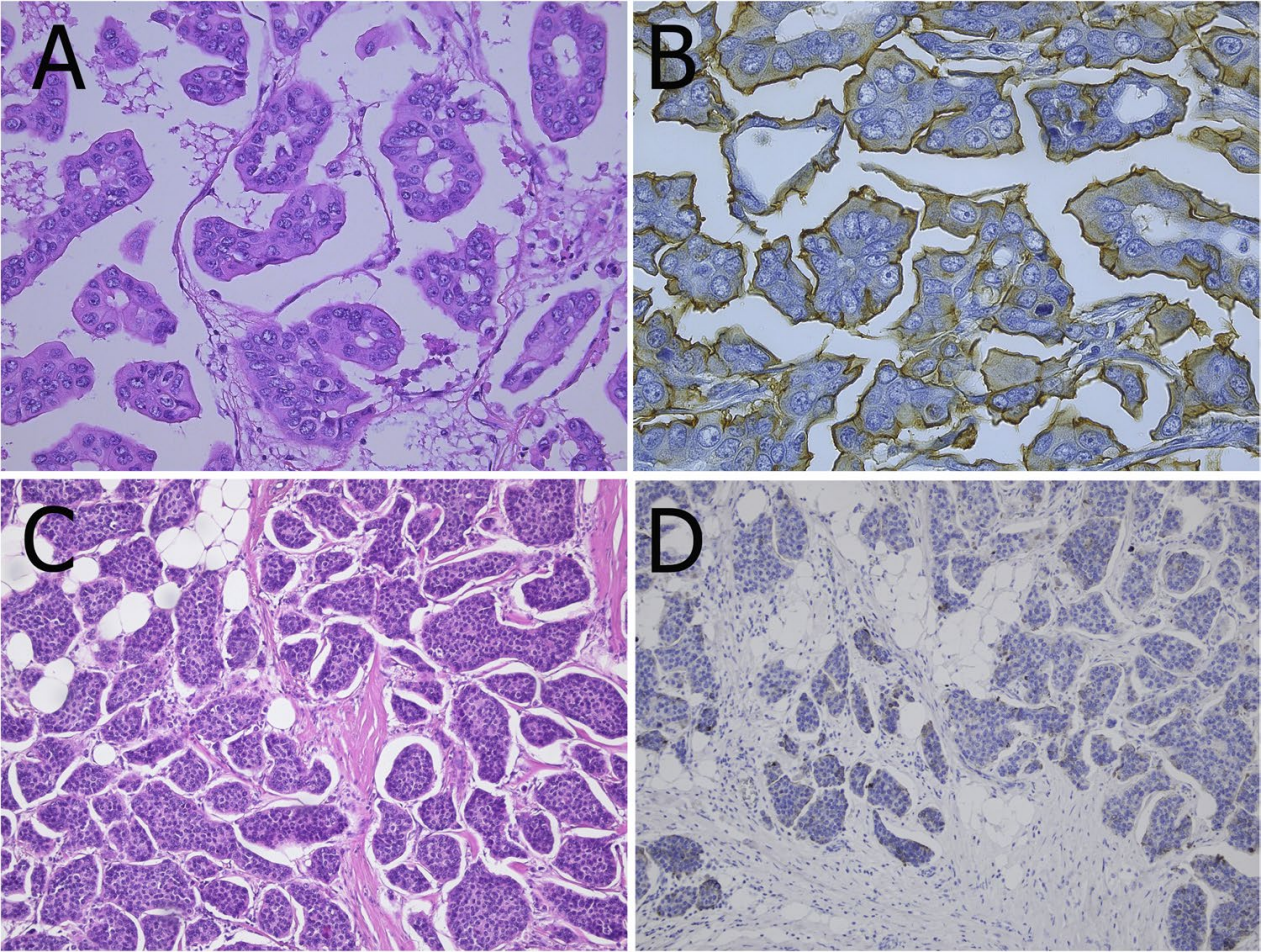
Invasive micropapillary carcinoma characterized by an “inside-out” growth pattern (**A**) highlighted by EMA immunoreactivity (**B**). In contrast, EMA is negative in invasive NST carcinomas with retraction clefts (**C, D**)

Invasive micropapillary carcinoma (IMPC) is characterized by clusters of cells within clear spaces showing an inside-out pattern. IMPC presents as palpable lesion with variable imaging features including frequent microcalcifications, all highly suspicious of malignancy [3, 6, 16]. With a prevalence of < 2%, pure IMPC is about four times rarer than mixed IMPC with NST [54]. Relatively small solid nests or rings of tumor cells are present in empty spaces because of detachment from the surrounding stroma mimicking retraction clefts, invasion in adipose tissue or in lymphatic vessels (Fig. 12) [2]. The apical pole is usually oriented towards the empty spaces displaying an”inside-out” or reversed polarity pattern, which can be highlighted by EMA or MUC1 immunohistochemistry [54]. Most IMPC are ER- and/or PR-positive. The reversed polarity is also reflected by the peculiar incomplete U-shaped basolateral membrane staining pattern for HER2, which challenges the current guidelines for the interpretation of HER2 immunohistochemistry [95]. Amplification of *HER2* is seen in 10–30% of IMPC, while a triple negative phenotype is rare. Despite higher frequency of lymph node metastasis, higher tumor grade, and more frequent lymph vascular invasion compared to NST carcinomas, pure IMPC does not show worse prognosis [16]. A higher level of stromal tumor infiltrating lymphocytes (TILs) seems to be associated with features of dismal prognosis when compared to IMPC with low TILs, being consistent with the predominant luminal phenotype of IMPC [22]. Immunohistochemistry for GATA3, WT1, and PAX8 is useful to exclude metastasis from ovarian serous carcinoma.

IMPC does not show pathognomonic mutations or translocations but distinctive complex patterns of copy number alterations as compared to NST carcinomas, such as 16q losses and 8q, 17q, and 20q gains [55]. Amplification of *MYC* (8q24*)*, *CCND1*, and *FGFR1* genes is frequent [56]. Mutations are present in the MAPK pathway and in *TP53* and *PIK3CA* [63]. Sporadic reports also describe mutations and deregulations in genes involved in cell polarity, shape, migration, and ciliogenesis [32].

### Secretory breast carcinoma with papillary growth pattern

**Fig. 13:**
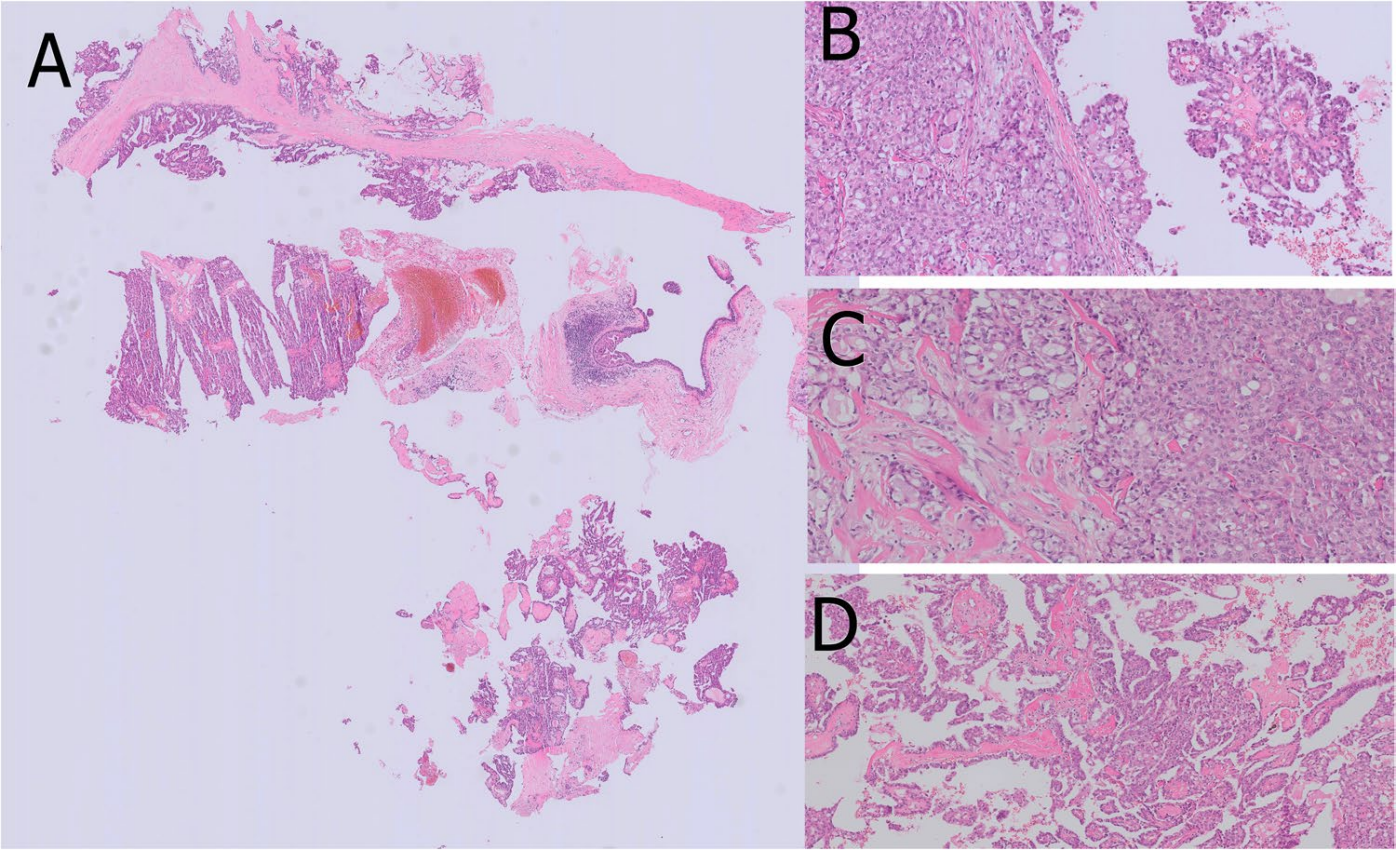
Secretory carcinoma with papillary architecture in a core needle biopsy (**A**). Solid areas (**B**) in transition to microcystic areas with presence of intraluminal eosinophilic secretion (**C**) and prominent papillary architecture (**D**) are typical cytoarchitectural features

Secretory breast carcinoma (SBC) is exceedingly rare and characterized by a pathognomonic recurrent *t(12;15)(p13;q25)* translocation, which results in the chimeric fusion gene *ETV6-NTRK3* [84]. SBC is mostly observed in post-menopausal women although it can occur at any age and also in males [36]. SBC presents as a slowly growing mass sharing radiological features with benign lesions like papillomas [87]. A predominant papillary pattern may be observed on occasion resulting in a challenging diagnosis on CNB [80]. SBC is composed of a heterogeneous cellular component including cells with amphophilic cytoplasm, apocrine aspect or a “bubbly aspect” due to abundant intracytoplasmic secretions (Fig. 13). Eosinophilic extracellular material positive for PAS, mucicarmine, and Alcian blue is consistently present. SBC usually shows a triple negative phenotype, but low ER expression and an ER − /PR + phenotype have been observed. S100 and mammaglobin are usually positive; expression of GCDFP-15 has been debated [18, 37, 47]. Pan-TRK immunohistochemistry has been suggested as a useful tool to confirm SBC diagnosis, or may be used for the selection of patients eligible for NTRK inhibitor therapy in the metastatic setting [79, 89]. The clinical course of SBC is indolent compared to IBC-NST; however, metastatic cases have recently been described [34, 37].

### Adenomyoepithelioma with papillary growth pattern

**Fig. 14:**
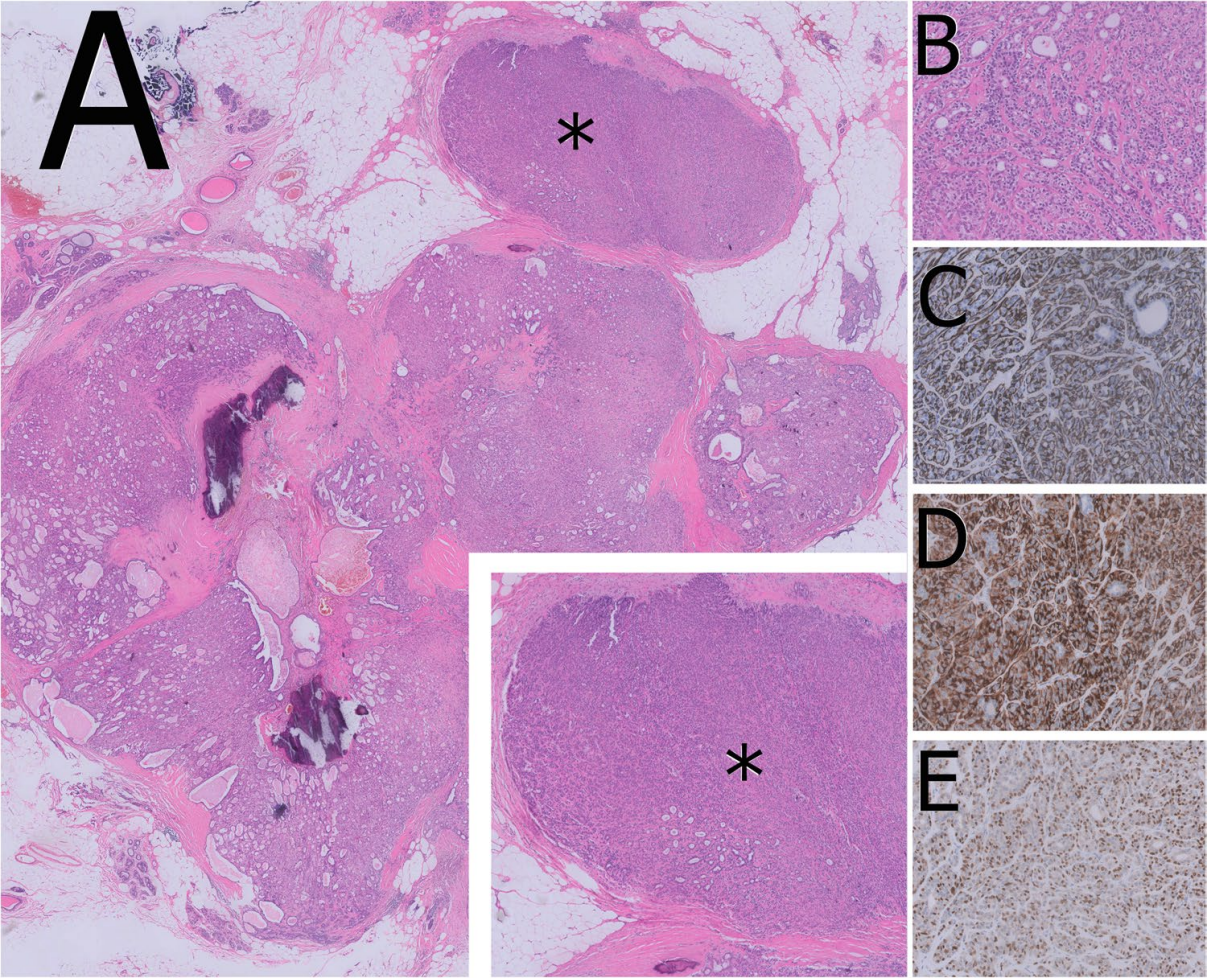
Adenomyoepithelioma with partial papillary growth pattern showing multinodular architecture with thick fibrovascular septa (**A**) and presence of expansive nodules of myoepithelial cells (*). The myoepithelial nodules show a mixture of glandular adenosis-like and spindle cell growth patterns (**B**) Proliferation of myoepithelial cells is confirmed by CK14 (**C**), calponin (**D**), and p63 immunostaining (**E**)

**Fig. 15:**
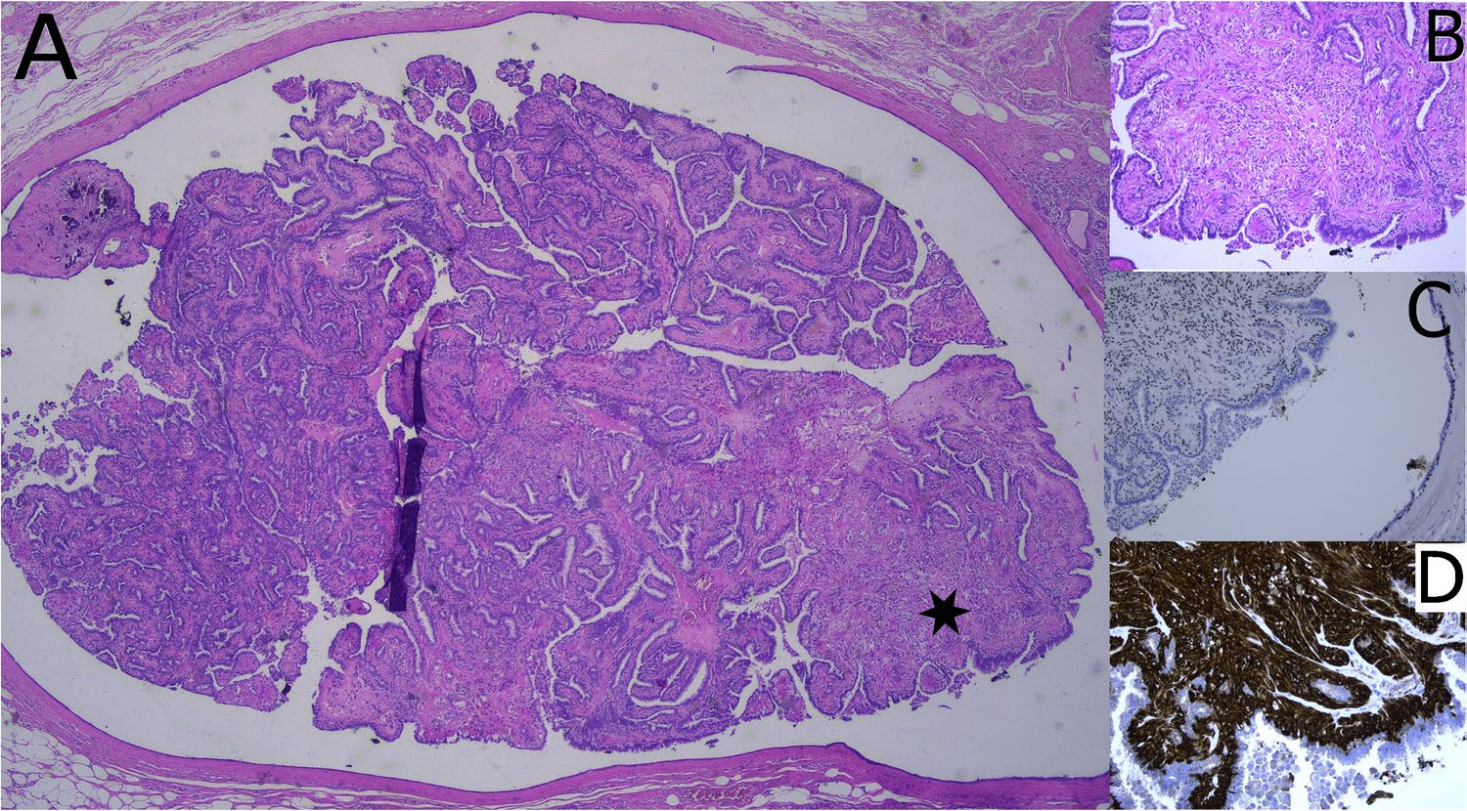
Intraductal papilloma with myoepithelial hyperplasia showing an area with increased cellularity (asterisk) (**A**) characterized by expansion of myoepithelial cells (**B**). The retained myoepithelial layer and the expansion of the myoepithelial compartment is highlighted by p63 (**C**) and CD10 (**D**) immunoreactivity

Adenomyoepithelioma (AME) of the breast is characterized by an epithelial-myoepithelial phenotype with heterogeneous architecture including a predominant papillary pattern [29]. AME is very rare and occurs mainly in post-menopausal women. Radiologically, it displays lobulated dense masses with often indistinct margins [57, 67]. AME diagnosis should be restricted to cases showing a biphasic cytology with prevalent expansion and proliferation of the myoepithelial component (Fig. 14). The distinction of AME from intraductal papilloma with myoepithelial hyperplasia is important due to the different biological behavior. While myoepithelial hyperplasia is generally focal in benign papillary lesions (Fig. 15), it is diffusely expanded in papillary AME [33]. AME can be ER-positive or -negative and is characterized by a different genomic landscape (e.g., *HRAS Q61* hotspot mutations in ER-negative AME) [30, 31]. The current WHO classification distinguishes between AME and malignant AME [27, 28]. However, the identification of atypical and malignant features is extremely challenging and remains a source of debate. Recently, the following detailed definitions for the distinction between AME and malignant AME were published [70]. Malignant AME in situ includes lesions with a classical AME architecture in which the epithelial component shows features of DCIS. The atypical cells show a cribriform or solid growth pattern with a well-defined margin or evidence of development within an intraductal-like structure. A peripheral myoepithelial cell layer at the epithelial stroma interface is typically seen. Malignant AME invasive (synonym: invasive adenomyoepithelial carcinoma) displays a dominant AME architecture but also has features sufficient for a diagnosis of malignancy including cytological atypia, increased mitotic activity, and necrosis associated with frankly invasive foci and an accompanying stromal response. The malignancy in these tumors can affect the luminal epithelial or the myoepithelial components or both [70].

### Nipple adenoma

**Fig. 16:**
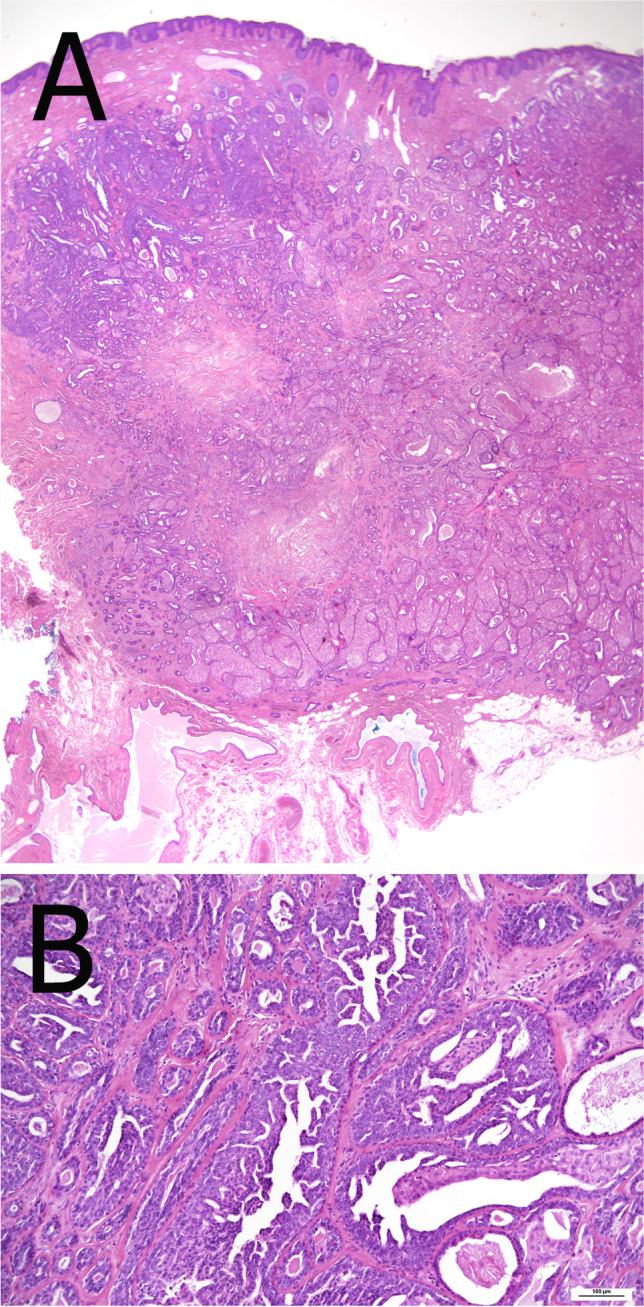
Nipple adenoma presenting as a mass at the dermo-epidermal junction with a large duct at the periphery (**A**). Focally, usual ductal hyperplasia with papillary architecture is evident (**B**)

Nipple adenoma (NA) is a benign tumor originally described as florid papillomatosis of the nipple [78]. NA occurs both in females and males with a wide age range (5 months to 89 years). Clinically, it may resemble Paget’s disease of the nipple. The dermo-epidermal junction is regarded as the site of origin. Involvement of the sub-areolar ducts with a general papillary aspect is a common feature. Histologically, a mixture of adenosis, papillary hyperplasia, and usual ductal hyperplasia is observed, frequently associated with squamous or apocrine metaplasia (Fig. 16). The presence of sclerosis may result in a pseudo-invasive growth pattern [78]. Immunohistochemistry using CK 5, ER, and myoepithelial markers helps in the differential diagnosis with DCIS or invasive carcinoma. The association with DCIS and invasive carcinoma has been rarely reported [1, 23, 91]. Mutations in *PIK3CA* are frequent; *K-RAS* and *BRAF* mutations may also occur [48]. Incomplete resection is associated with recurrence.

### Non-neoplastic lesions with papillary structures

A variety of non-neoplastic lesions may show a papillary or micropapillary pattern. Among those, juvenile papillomatosis, florid gynecomastia with micropapillae-like intraductal epithelial proliferation, papillary apocrine metaplasia and UDH with papillary pattern (papillary intraductal hyperplasia) are encountered. These lesions are beyond the scope of this review and are not further discussed in detail.

## Conclusion

Papillary breast lesions form a heterogeneous group of neoplastic and non-neoplastic diseases of which some may cause diagnostic difficulties. Immunohistochemistry, particularly for myoepithelial markers, is helpful for differential diagnosis. Triple negative carcinomas with papillary architecture are rare and considered non-aggressive TNBC. Due to increasing experience and endeavor to avoid overtreatment, the clinical management of benign intraductal papilloma seems to become more conservative, while keeping strict criteria of eligibility for non-operative treatment. In this respect, increasing knowledge about molecular genetic alterations will help to optimize therapeutic strategies.
